# A comparative assessment of contamination rates in gastrointestinal endoscope reprocessing: sterilization versus high‐level disinfection

**DOI:** 10.1002/deo2.70093

**Published:** 2025-03-09

**Authors:** Tanyaporn Chantarojanasiri, Rachanikorn Rungrueangmaitree, Siriporn Thongsri, Urasa Jampa‐ngern, Thawee Ratanachu‐Ek

**Affiliations:** ^1^ Department of Internal Medicine Rajavithi Hospital, College of Medicine, Rangsit University Bangkok Thailand; ^2^ Department of Internal Medicine Charoenkrung Pracharak Hospital Bangkok Thailand; ^3^ Department of Surgery Rajavithi Hospital Bangkok Thailand

**Keywords:** bacterial contamination, duodenoscope, endoscope reprocessing, endoscopic ultrasound, high‐level disinfection, sterilization

## Abstract

**Objectives:**

This study aimed to evaluate the disparity in culture results between sterilization and high‐level disinfection (HLD) for duodenoscopes and linear endoscopic ultrasound (EUS), and to assess the effectiveness of different bacterial contamination detection methods.

**Methods:**

This is a prospective randomized study, including duodenoscopes and linear EUS with adenosine triphosphate bioluminescence assay values below 200 relative light units after manual cleaning which were randomly assigned to undergo either sterilization or HLD in a 1:1 ratio. Following disinfection, all endoscopes were subjected to adenosine triphosphate bioluminescence assay testing and cultures using both swab and liquid samples from endoscope channels.

**Results:**

Totally 752 endoscopes (444 duodenoscopes and 308 linear EUS) were studied. After disinfection, the positive culture rates for the sterilization and HLD groups were 5.9% and 7.2%, respectively (*p* = 0.460). No significant difference in contamination rates was observed between duodenoscopes and linear EUS (5.9% and 7.5%, respectively; *p* = 0.379), and no significant association between contamination rates and the presence of biliary stones was seen (7.3% vs. 6.9%; *p* = 0.613). The detection rate of bacteria from liquid samples taken from endoscope channels was 0.5%, which was significantly lower than the swabbing method (6.0%, *p* < 0.001).

**Conclusions:**

This study found no statistically significant difference in contamination rates between sterilization and HLD methods for gastrointestinal endoscope reprocessing. The type of endoscope and the presence of biliary stones did not influence the positive culture rate. The swabbing method showed significantly higher bacterial detection when compared with liquid samples.

## INTRODUCTION

Flexible endoscopes used in gastrointestinal endoscopy are classified as semi‐critical medical devices that contact with mucous membranes and might act as the vector for human‐to‐human transmission.^1^, ^2^, ^3^, ^4^, ^5^ Endoscope reprocessing is one of the most important steps to prevent these endoscope‐transmitted infections to remove soil and eliminate microbial bioburden in these reusable endoscopes.^6^ The minimal standard for reprocessing is high‐level disinfection (HLD),^7^ which destroys all types of vegetative non‐spore‐forming bacteria (Gram‐positive and Gram‐negative), fungi, all types of viruses (hydrophilic and lipophilic), and mycobacteria.^3^ On the other hand, sterilization destroys all microorganisms including highly resistant bacterial spores.^1^, ^7^ The HLD technique included pre‐cleaning, manual cleaning, and reprocessing in automated endoscope reprocessors where the endoscope was soaked in an HLD disinfectant at the labeled exposure time and temperature as recommended by the company.^7^ The sterilization technique contains similar steps for the cleaning as HLD as described above, but the exposure time for sterilization is longer than for disinfection in order to kill spores and spore‐forming bacteria.^8^ Various sterilization methods have been proposed, such as ethylene oxide (ETO) gas sterilization, Hydrogen peroxide gas sterilization with and without plasma, and low‐temperature steam and formaldehyde sterilization.^9^ Another liquid chemical sterilization included sterilization using Peracetic acid, which is more powerful than using glutaraldehyde or orthophthalaldehyde.^10^ Peracetic acid is a highly biocidal oxidizer that removes surface contaminants (primarily protein) and has been used for the sterilization of medical and dental instruments in automated machines. It has the ability to inactivate gram‐positive and gram‐negative bacteria, fungi, yeasts, viruses, and bacterial spores when the equipment is exposed at different times and concentrations.^11^

European Society of Gastrointestinal Endoscopy‐European Society of Gastroenterology Nurses and Associates (ESGE‐ESGENA) guideline in 2007 stated standard testing of the endoscope which includes collection of liquid samples from endoscope channels for the bacterial culture and non‐culture method.^12^ As routine bacterial cultures take around 3–5 days for incubation, non‐culture methods such as Adenosine triphosphate (ATP) bioluminescence assay have been used more frequently as it provides a rapid measurement for micro‐organisms within a few minutes.^5^, ^13^ This technique estimates the ATP level, which is present in microorganisms, by using the light‐producing reaction between ATP, luciferin, and luciferase.^13^ Luminometers convert the number of photons released in the reaction into relative light units (RLUs).^14^ However, ATP has limitations in discriminating between residual microbes, cellular debris, or organic soil and cannot replace the routine bacterial culture.^15^

Although the sterilization technique should be safer than the standard HLD, it requires more time to process which results in a longer endoscope turnover rate. Moreover, there have been limited studies regarding the clinical significance of these two.^12^ The main objective of this study was to compare the contamination rate between sterilization and high‐level disinfection for gastrointestinal endoscope reprocessing. Secondary objectives were to evaluate the detection methods for the contamination as well as other factors that determine the contamination rate.

## METHODS

**FIGURE 1:**
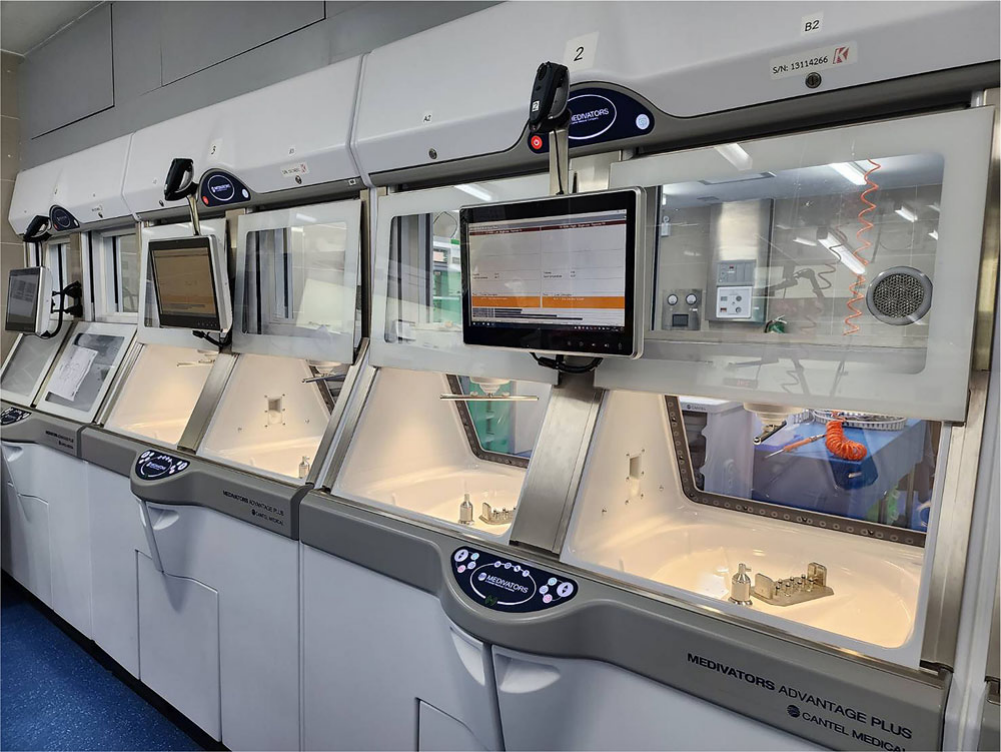
The ADVANTAGE PLUS Pass‐Thru Automated Endoscope Reprocessor (CANTEL Medical Corp.) machine.

This was a prospective randomized study including duodenoscopes and linear echoendoscopes the ATP level after manual cleaning was less than 200 RLUs.^16^ After manual cleaning, endoscopes were randomized to be disinfected using automated endoscope reprocessors either by sterilization or HLD technique in a 1:1 ratio. Reprocessing methods were performed with ADVANTAGE PLUS Pass‐Thru Automated Endoscope Reprocessor (AER; CANTEL Medical Corp.; Figure 1) for both sterilization and HLD group, using peracetic acid as the disinfectant. For sterilization, we used peracetic acid at concentrations of 850 ppm, at room temperature, and kept contact time with the endoscope for 10 minutes while in In HLD group, peracetic acid at the concentrations of 850 ppm, room temperature, was kept in contact for 5 minutes. As the contamination in the AER is common, mostly from contaminated rinsing water,^17^, ^18^ water used in our AER underwent reverse osmosis before entering the machine.

**FIGURE 2:**
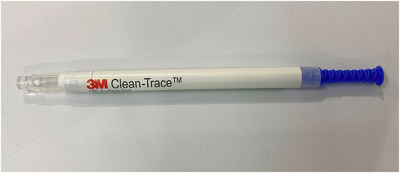
Adenosine triphosphate bioluminescence assay test kit using 3 M Clean‐trace surface ATP (3 M Clean Trace).

After disinfection in an automated endoscope reprocessor, all endoscopes were evaluated for ATP and cultures. For ATP tests, we obtained samples by swabbing the elevator mechanism with 3 M Clean‐trace surface ATP (3 M Clean Trace; Figure 2) and measuring with 3 MClean‐trace luminometer (3 M Clean Tract) which displayed the test result in RLU. Specimens for culture were obtained from endoscope channels using swabs and liquid samples. For the swabs method, sterile brushes were rotated over the endoscopic elevator both in closed and open positions before being sent to bacterial culture in a sterile container. For liquid samples, 20 mL of sterile saline was flushed through the suction and working channels of each endoscope and collected in sterile containers before sending for bacterial culture.

**FIGURE 3:**
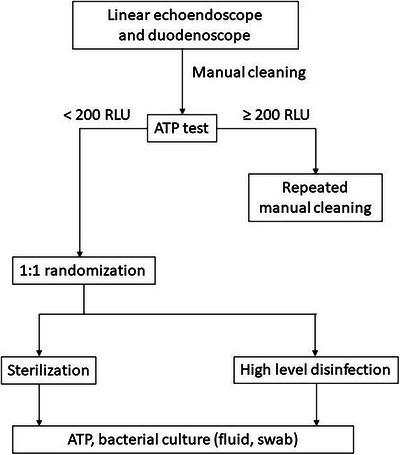
Flowchart demonstrates the study process.

Liquid samples from endoscope channels were incubated at 30°C on Tryptic soy agar, brain heart infusion broths, and thioglycollate broth. Samples from the swab method were also incubated at 30°C in grain heart infusion (broths and Thioglycollate broth. The final report for negative culture tests was 7 days of incubation. Figure 3 demonstrates the flowchart of this study.

As the endoscopic procedure related to biliary stones might associated with bacterial infection, the presence of biliary stones and sludges as well as pancreatic duct stones in the endoscopic report were recorded and analyzed.

### Outcomes

The primary outcome was to compare the contamination rate as determined by the difference of culture results between sterilization and high‐level disinfection. Secondary outcomes were the sensitivity and specificity of ATP bioluminescence assay for detecting microorganisms and the effect of the sampling method. Clinical factors associated with contamination rate were also analyzed.

### Statistical analysis

The sample size was calculated based on the study from Larsen that reported the incidence of contamination rate after duodenoscope reprocessing with HLD of 0.16 and that after double HLD ETO gas sterilization of 0.09.^19^ When assuming a type I error of 5% (*p* < 0.05) and a type II error of 0.20 (80% power) a total sample size of at least 728 endoscopes needed to be enrolled in the study.

The categorical data was reported as the number and percentage. The continuous data variables were presented as a mean ± standard deviations. The continuous data were compared using the student's *t*‐test, and the categorical data were compared using the chi‐square test and McNemar's test. Statistical significance was considered significant at *p*‐value < 0.05 (2‐sided). All statistical analyses were performed by using IBM SPSS Statistics for Windows, version 22.0.

## RESULTS

### Baseline characteristics of the endoscopes

Overall, 752 endoscopes in which ATP test after manual cleaning <200 RLU, which associated with complete manual cleaning,^20^, ^21^ were included. Among these, 444 (59.0%) were duodenoscopes and the remaining 308 endoscopes were linear echoendoscope. The indications include biliary drainage and stricture management in ERCP, diagnostic and tissue acquisition as well as EUS interventions in EUS procedures. Stone and sludges were presented in 302 cases (40.16%) and 237 cases (31.52%) of duodenoscope. In the remaining 65 cases of EUS in the presence of stone, EUS was performed before ERCP for the detection of choledocholithiasis. We random 376 endoscopes to the HLD group and the remaining 376 endoscopes to the sterilization group. The baseline information of endoscopes and reprocessing methods are shown in Table 1.

**TABLE 1:** Type of endoscopes, the presence of stone/sludge, reprocessing methods, adenosine triphosphate bioluminescence assay (ATP), and Culture results.

	Culture results		
Factors	Positive (%) (*n* = 49)	Negative (%) (*n* = 703)	*p*‐value
Reprocessing methods			0.460
HLD	27 (7.2)	349 (92.8)	
Sterilization	22 (5.9)	354 (94.1)	
Types of endoscopes			0.379
Duodenoscope	26 (5.9)	418 (94.1)	
Linear EUS	23 (7.5)	285 (92.5)	
Stone and sludge (All endoscopes)			0.613
No	31 (6.9)	419 (93.1)	
Yes	18 (7.3)	284 (92.7)	
Stone and sludge (duodenoscopes)			
No	12 (5.8)	195 (94.2)	
Yes	14 (5.9)	223 (94.1)	
ATP (RLUs)	67.71 ± 49.08	64.02 ± 44.72	0.963

Abbreviations: EUS, endoscopic ultrasound; HLD, high‐level disinfection.

### Culture results

The overall positive culture in this study was 6.5%. The most common microorganisms were *Enterococcus faecalis* (20.4%), *Bacillus cereus* (14.3%), *Staphylococcus epidermidis* (12.2%), and *Staphylococcus warneri* (10.2%; Table 2). When comparing both reprocessing methods, the sterilization group had a 5.9% positive culture result, which was lower than that of the HLD group (7.2%). However, this difference was not statistically significant (*p* = 0.460; Table 1). Considering other factors that might affect the result of reprocessing, there was no significant correlation between positive culture results and type of endoscope and the presence of stones and sludges in the duodenoscope, with *p*‐values of 0.379 and 0.961, respectively (Table 1). The types of organisms detected in each type of reprocessing as well as the detection method are described in Table 2.

**TABLE 2:** Prevalence and types of organism detected according to reprocessing method and sampling techniques.

Reprocessing method	HLD		Sterilization		No. of positive culture (%) (*n* = 57)^*^
Detection method	Fluid	Swab	Fluid	Swab	
*Enterococcus faecalis*		6		4	10 (17.54)
*Bacillus cereus*		4		3	7 (12.28)
*Staphylococcus epidermidis*		3		3	6 (10.53)
*Staphylococcus warneri*		4		1	5 (8.77)
*Escherichia coli*		1		1	2 (3.51)
*Klebsiella pneumoniae*	1		1		2 (3.51)
*Micrococcus luteus*			1	1	2 (3.51)
*Morganella morganii*		1		1	2 (3.51)
*Staphylococcus haemolyticus*		1		1	2 (3.51)
*Staphylococcus hominis*		1		1	2 (3.51)
*Staphylococcus pasteuri*		1		1	2 (3.51)
*Aerococcus viridans*				1	1 (1.75)
*Brevundimonas diminuta*				1	1 (1.75)
*Bacillus pulminis*				1	1 (1.75)
*Bacillus subtilis*		1			1 (1.75)
*Cellulosimicrobium cellulans*		1			1 (1.75)
*Cutibacterium spp*		1			1 (1.75)
*Enterobacter cloacae*				1	1 (1.75)
*Moraxella osloensis*			1		1 (1.75)
*Roseomonas mucosa*		1			1 (1.75)
*Rhizobium radiobacter*			1		1 (1.75)
*Rhizobium* spp.				1	1 (1.75)
*Serratia marcescens*		1			1 (1.75)
*Staphylococcus capitis*				1	1 (1.75)
*Stenotrophomonas maltophila*				1	1 (1.75)
Mold				1	1 (1.75)

Values are represented as *n* (%).

Abbreviation: HLD, high‐level disinfection.

* Multiple organisms in the same scope were counted separately.

When compared between the culture sampling methods, liquid samples from endoscope channels resulted in positive culture in four out of 752 cultures (0.5%), which is significantly lower when compared with positive tests in 45 out of 752 culture samples (6.0%) in the swab method (*p* < 0.001; Table 3.).

**TABLE 3:** Different sampling techniques and the positive culture result.

Sampling methods		Culture results (%)		
	Total	Positive	Negative	*p*‐value
Fluid	752 (100.0)	4 (0.5)	748 (99.5)	<0.001^*^
Swab	752 (100.0)	45 (6.0)	707 (94.0)	

### The ATP tests

Apart from the swab culture, we performed the ATP test from the elevator mechanism of all endoscopes. The mean ATP after endoscope reprocessing was comparable in both cultures: culture‐positive group (67.7) and culture‐negative group (64.0; *p* = 0.963; Table 2). The area under the curve for positive bacterial contamination was 0.484. When we used the ATP cut‐off value at 40 RLU as described in the previous study,^18^ the sensitivity and specificity for positive bacterial culture were only 59.2% and 39.7%, respectively.

### Clinical follow‐up

No outbreak of endoscopic transmitted infection was observed during the study period. Among 46 cases with a positive culture, there were two patients with post‐ERCP cholangitis including one with mild cholangitis and one with severe cholangitis. In a case with severe cholangitis, the patient developed Klebsiella septicemia which is different from Bacillus spp. which was identified from the swab.

## DISCUSSION

We performed the study comparing the efficacy of high‐level disinfection and sterilization using peracetic acid as the disinfectant.^13^ Our study, which compared the decontamination rate between HLD and sterilization in 752 endoscopes (444 duodenoscopes and 308 linear EUS), showed no significant difference in positive culture result (*p* = 0.46). To control the initial degree of contamination, we excluded the cases in which ATP level after manual cleaning more than 200 RLU since this reflects the retaining residual organic and bioburden.^20^

There have been several studies comparing HLD and other methods such as double HLD or using ETO gas for decontamination.^19^, ^22^, ^23^, ^24^ A systematic review and meta‐analysis by Larsen,^19^ demonstrated that the contamination rate after only using HLD was 16.14% ± 0.019 (95% CI: 12.43%–19.85%) which is higher than after using either double HLD or ETO gas sterilization (9.2% ± 0.025 [95% CI: 12.43%–19.85%]).^19^ When using ETO gas sterilizations of duodenoscopes,^22^ the rate of positive culture after ETO gas sterilization was 1.2%, which was improved when compared with the reported level of 2% after HLD.^25^ Our study used peracetic acid as the disinfectant, which resulted in a 7.2% bacterial detection rate after disinfection. This is much lower than the 16.14% detection rate in the previous meta‐analysis.^19^ The possible explanation for these differences in the detection rate is that the meta‐analysis included studies performed in various centers between the years 2010–2020. On the contrary, our study only included those using the automatic reprocessing machine, which is now currently recommended as the standard reprocessing process.

We studied other factors that might affect the contamination rate. As most cases of ERCP deal with biliary stones, which are considered contaminated compared to EUS, the rate of positive culture observed from the duodenoscope and linear EUS were analyzed. The contamination rate between the overall duodenoscope and linear EUS did not show a statistical difference [5.9% and 7.5%, respectively, (*p*‐value 0.379)]. When focusing only on the duodenoscope with or without biliary stones, there was no significant difference in the rate of positive culture in the stone group (5.9%) and the no‐stone group (5.8%). As these scopes presented variable levels of contamination, we tried to eliminate the baseline difference in bacterial load by excluding those with ATP levels above 200 RLU after manual cleaning and using the water that underwent reverse osmosis to eliminate the water‐borne contamination. The species identified in the culture included *S. epidermidis* and *S. warneri*, which are common bacterial contaminants. Still, two cases of acute cholangitis after ERCP were observed during our study but the pathogen identified was not the same as the culture from the scope tip.

Our study also underlined the technique to detect endoscopic contamination. The liquid sample method showed a significantly lower rate of positive bacterial culture when compared with the swab method (0.5% vs. 6.0%, *p* < 0.001). This finding is similar to the study by Cattior which showed superior results when the brush is used for detecting bacterial contamination.^26^ Also, our study did not show significant differences in ATP levels among those with positive and negative bacterial cultures which is similar to the previous studies.^25^, ^27^ When using an ATP cut‐off level of 40 RLU as recommended by the study by Ridtitid,^28^ we demonstrated only 59.2% sensitivity and 39.7% specificity. These findings emphasized the importance of the method to detect bacterial contamination.

There have been several studies comparing various methods of endoscopy decontamination but still limited studies directly comparing the contamination rate between sterilization and HLD. However, there are some limitations as this study has been conducted only in a single center. Moreover, the neutralizer that is used in many studies is not available in our country, so we only use sterile water that is transferred immediately to the laboratory room for microbial incubation. Further studies in various centers should be conducted to determine the benefit of sterilization over HLD.

In conclusion, our study did not find any significant difference in bacterial contamination rates between reprocessing using HLD and sterilization. Adding time for sterilization did not show a clinical advantage over standard HLD. These results support the recommendation of the ESGE‐ESGENA guidelines,^7^ which suggest that reprocessing with HLD is a standard method currently used for the disinfection of endoscopes. Further studies are needed to confirm the validity of ATP for detecting microbial contamination in gastrointestinal endoscopes.

## CONFLICT OF INTEREST STATEMENT

None.

## ETHICS STATEMENT

Approval of the research protocol by an Institutional Reviewer Board: N/A

## PATIENT CONSENT STATEMENT

N/A.

## CLINICAL TRIAL REGISTRATION

N/A.
